# Prevalence of depression among elderly women in India-An intersectional analysis of the Longitudinal Ageing Study in India (LASI), 2017-2018

**DOI:** 10.21203/rs.3.rs-2664462/v1

**Published:** 2023-03-14

**Authors:** Paramjot Panda, Prashansa Dash, Manas Behera, Trupti Mishra

**Affiliations:** AIPHU; KIT Deemed to be University; KIT Deemed to be University; UNICEF

## Abstract

Epidemiological transition in India shows a shift in disease burden from youth to the elderly. As Life Expectancy increases, a greater burden is placed on the state, society, and families in India. Mental health disorders are insidious, debilitating Non-Communicable Diseases (NCDs) that afflict people, their families, and generations down the line. Globally, depression is the leading cause of mental health-related disability. It is estimated that mental illness contributes to 4.7% of Disability Adjusted Life Years (DALYs) in India. It is predicted that by 2026, the elderly's sex ratio will increase to 1,060 feminizing ageing. Research has shown that elderly women in developed countries like the United States are more prone to depression. Chronic morbidities are more common in women than in men, and they may suffer from poor vision, depression, impaired physical performance, and elder abuse. Mostly widowed, economically dependent, lacking proper food and clothing, fearing the future, and lacking proper care, they have difficulty coping with these health problems. There are surprisingly few studies on elderly female depression. Therefore, we want to hypothesize the prevalence of depression among women in different regions and demographic groups in India, and what factors may contribute to these differences. Using intersectional analysis with the data from Wave 1 (2017-2018) of the (Longitudinal Ageing Study in India) LASI (N = 16,737) we were able to explore the intersecting patterns between different variables and how people are positioned simultaneously and position themselves in different multiple categories based on the type of place of residence, age and level of education. Through the study we further aim to determine the prevalence of depression among elderly female in the age group of 60 in different states using the Chloropleth map. The findings of the study highlight the significance of the place of residence in the development of depression among elderly women, with the rural area being associated with a higher prevalence of depression compared to urban area. When compared to people with higher literacy, those with low literacy were significantly associated with depression. State-wise, there is a huge difference between the prevalence of elderly women depression in rural and urban areas. The study highlights the vulnerability of elderly women to depression. It is possible for the government to develop programs that address the needs of elderly women, both in urban and rural areas, to reduce depression. Multi-factor approaches to mental health, which consider age, literacy, and location, are essential. Programs targeting specific populations can be developed to address depression's root causes..

## Introduction

People and their families, and generations down the line, suffer greatly from mental health disorders, an insidious, often debilitating form of Non Communicable Diseases(NCDs)^1^. The greatest mental health-related burden is attributed to depression, which is a leading cause of disability worldwide^2^. Mental health impairment worsens a number of NCDs risk factors, such as poor lifestyle choices resulting in obesity, inactivity, and tobacco abuse, poor health literacy, and a lack of access to health promotion activities^3-5^. As per the WHO Global Burden of Disease Report globally, 4.4% of the population is estimated to be depressed in 2015 ^6^ . The report also says that around the world, 322 million people are suffering from depression ^7^. **There is a higher incidence of depression among females (5.1%) than among males (3.6%).** It is estimated that this number is growing each year. Even though women live longer than men, research shows they are more prone to certain diseases, which can ultimately shorten their lifespan^8^. Strokes, depression^9^, Alzheimer's^10^, and autoimmune diseases^11^ such as multiple sclerosis and rheumatoid arthritis^12^ are among them. Most of these people live in the South-East Asia Region and the Western Pacific Region, due to the fact that these regions have much larger populations than the others (which include India and China, for example)^13-15^. According to estimates from 2015, the number of Years Lived with Disability (YLD) related to depression amounted to more than 50 million around the world^7^. In terms of the prevalence of non-fatal health loss, depression disorders are ranked as one of the most significant contributors (7.5% of all YLD) ^6^.

As per the WHO report for low- and middle-income countries, depression poses a significant public health challenge due to its comorbidity with chronic physical disease ^16^. It is estimated that depression has a 2-4-fold higher prevalence in patients with chronic diseases such as cancer, diabetes mellitus, stroke, or cardiovascular disease, and the disease may last longer ^16^. Mental disorders such as depression and anxiety contribute to the escalation of non-communicable diseases through non-adherence to treatment ^17^. Those with mental disorders may have a harder time accessing healthcare, treatments may require behavioural changes that may be harder for them. Stigma associated with mental disorders is identified as a barrier to access the health facilities ^3,18^. According to the findings of a population-based study^19^, it was found that older people with multimorbidity are more likely to experience depressive symptoms in later life ^19^. Researchers have identified that functional health factors acts as a mediator between multimorbidity and depression, especially when it comes to older women and very few have reported it ^20^. Also most of the researches have recommended further longitudinal research to understand functional and behavioural health in multimorbidity-depression relationships^20-22^. The WHO says depression is 50% more prevalent in women than in men, and Indians are among the most depressed worldwide^18^

As India is the most populous country and the largest democracy, is now emerging as the sixth-largest economy in the world. It is recently seeing a demographic transition with increase in elderly population. In accordance with the United Nations definition of a "Graying Nation", a country is defined as a greying country where the percentage of people who are over 60 years of age is at least 7% of its total population^23^. There were almost 7.7% of people in India, at the dawn of the millennium, were old, and this figure increased to 8.6% in 2011, and 9.4%, in 2017 ^24^. Also most of the researcher have forecasted by seeing the trend that by the year 2050, there will be 20 % of the elderly people (almost 300 million) ^25-27^.

From 2011 through 2041, India is forecast to gain a demographic advantage due to a larger proportion of the population in the working age group ^25^. And after 2041, when the aging burden shall begin, the older population may contribute to second demographic growth by accumulating capital from their savings accumulated during their working years ^28^. But this depends on developing financial markets, a healthy older population, and social security, all of which seems to be daunting at the moment. Additionally, due to the epidemiological transition, a large portion of the burden of disease has been shifted from the youth to the elderly^29^. Non Communicable Diseases (NCDs) exceeded 50% in the 30–34 age group and were highest at 78.8% in the 65–69 age group ^29^. An increasing Life Expectancy can be attributed to increased longevity and growing society, but it can also be attributed to an increased demand for healthcare facilities, placing an increased burden on the state, society, and families in India^19^. Out-of-pocket health expenses account for more than 70% of health expenditures in India, leaving the older population vulnerable to health problems ^30^.The Disability Adjusted Life Year (DALY) rate between 1990 and 2016 was the highest for diseases such as diabetes (80.0%), ischaemic heart disease (33.9%), and sense organ diseases (mainly vision and hearing loss disorders 21.7%) ^31^.

In India, mental illness is prevalent and pervasive, especially among older adults living in a distressed socioeconomic situation ^29^. Researches have reported due to the social stigma of mental illness in older adults and the lack of trained mental health professionals, the prevalence of mental illness among older adults is higher than the reported figures ^32,33^.There were 197 million people in India who lived with a mental disorder in 2017. Of those people, 45 million suffered from depression and another 44 million from anxiety ^34^. Mental disorders are a major contributor to the total number of DALYs in India, and their share increased from 2·5% in 1990 to 4·7% in 2017^34^. There is a high incidence of depression among the elderly population of India with females being predominant in the group ^35^.

As per the census 2011^36^ the majority of older Indian adults live in rural areas, over 70% of whom are illiterate, while over half of them do not have a source of income ^37^. Quacks, folk healers, or AYUSH practitioners provide health and mental health care to older adults in rural areas, since allopathic doctors and hospitals are far away in urban areas and elderly people often have difficulty approaching hospitals ^38^. Though there are scarce research publications available related to depression among elderly population in rural area, but those available shows that as a result of different population characteristics, depression prevalence is slightly but significantly higher in rural areas than in urban areas ^39^. However, these studies are conducted in a small population. In a study in South India it was revealed that the prevalence of depression varies among rural and urban area^35,40^.

In India National Mental Health Policy,2014 aims to reduce distress, disability, exclusion morbidity and premature mortality associated with mental health problems across the life-span of a person ^41^. Nevertheless, a larger part of the policy is focused on ensuring the mental health of the population as a whole, with little emphasis on mental health of the elderly ^42^. The reality is, with the presence of mental health disorders and comorbid conditions, the elderly population is more likely to suffer from mental health problems, contributing to a larger burden of dual disease in the country as a whole ^43^. Though mental disorders are studied in different parts of India, including in the National Mental Health Survey there has been very limited resource available which highlights the prevalence of state-wise and gender-wise depression its association with disability-adjusted life years (DALYs).

Researches have revealed that female elders are more likely to suffer from physical and mental disabilities that greatly reduce their quality of life^44^. Studies in the United States have shown that elderly women are more susceptible to depression, experience longer and more persistent depression, and have lower mortality rates once depressed^44,45^ . There is no doubt that late-life depression poses a significant public health problem since it is widespread and expensive, associated with disability, re-hospitalization, and even death among those with chronic diseases^46,47^. According to the literature Compared to elderly men, women are more likely to suffer from chronic morbidity^48^, poor vision^49^, cataracts, high blood pressure, back pain/slipped disk, malnutrition^50^, depression^10^, impaired physical performance^51^, and elder abuse^52^, women have difficulty coping with these health problems because they are widowed, economically dependent, lack proper food and clothing^15^, fear the future, lack care, and suffer from progressive health decline ^53^. However, existing policies and programmatic capacities are inadequate and lack gender sensitivity to address the socioeconomic and health needs of women. By utilizing secondary data from Longitudinal Ageing Study of India (LASI), this study fills a gap by identifying the prevalence of common mental disorders like depression in elderly females in India by identifying a number of factors related to it, especially depression, in females in India and its trend with respect to age, economic status, place of residence, marital status, alcohol consumption, tobacco consumption, and physical activity. The study also shows its trend in urban as well as rural areas. It will provide valuable insight to policymakers so that they can develop the necessary policy implications to address the rapidly increasing rate of depression among the female elderly population in India.

### Aim and Objective:

What is the prevalence of depression among women in different regions and demographic groups in India, and what factors may contribute to these differences?

- To determine the extent of depression among women in various regions and demographic groups in India.
- To determine the factors associated with depression among the elderly women in India.

## Results

Their distribution was calculated using descriptive statistics by gender, age, place of residence, and education status. Binary Logistic Regression was used to estimate the prime factors associated with depression since depression was a binary yes or no variable. Unadjusted odd ratio was obtained through the first regression model to control other variables. Our second regression model takes into account other variables such as age, place of residence, marital status, education, employment, household income, alcohol, tobacco, physical activity, and yoga to determine the risk factors for depression. Using intersectional analysis, we explored how people are positioned simultaneously according to their place of residence, their age, and their level of education, as well as how they position themselves in different multiple categories. To understand the distribution of depression prevalence among Indian states and Union Territories, we have plotted the Choropleth map using the GeoDa software.

The study collected data on various variables such as age, place of residence, marital status, education, employment status, household income, alcohol consumption, tobacco consumption, physical activity, and depression. Age was categorized into four groups, and place of residence was categorized into rural and urban areas. Marital status was categorized as a nominal variable, and education status was based on the number of years of schooling. Employment status was reported as a nominal variable with Yes for those who were currently working and No for those who were not. Household income was categorized into five groups. Alcohol and tobacco consumption were captured as nominal variables with Yes for those who consumed them and No for those who did not. Physical activity and yoga were reported as a nominal variable with Yes for those who did them regularly and No for those who did not. The study's outcome variable was depression, which was assessed using the Composite International Diagnostic Interview-Short Form (CIDI-SF) scale, with Yes for those who had depressive symptoms and No for those who did not. The study ensured reliability and validity by training non-clinicians to collect data using the CIDI-SF tool in the local language.

Table 1 The study involved 14553 women who were 60 years old or older, and those below 60 were excluded from the analysis. The percentage of participants in the 60-64 age group was 33.6%, while the proportion decreased as age increased, with women over 75 accounting for 24.4%. This could be due to increased life expectancy and better access to healthcare. The majority of participants (63.8%) were from rural areas, and most (90.2%) were married. Nearly half (46.2%) had some basic education, while 31.6% had completed 10 or more years of education. The majority (60.7%) of the women were employed. Household income was distributed equally across most wealth categories. Only a small percentage of participants consumed alcohol (3.9%), while 19% used tobacco and only 12.2% engaged in regular exercise.

### Geoda Software

**Figure 1:**
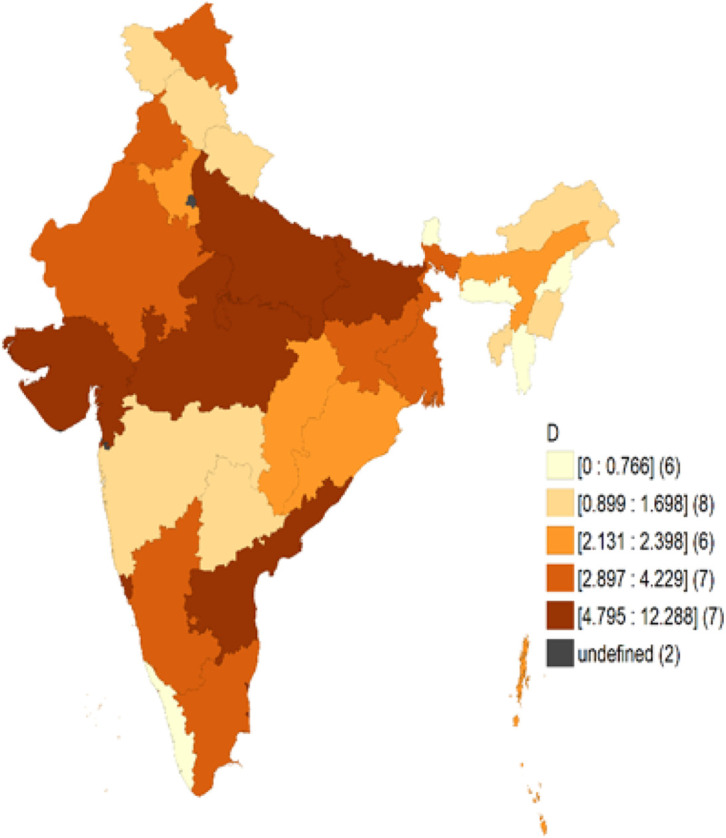
state wise Prevalence of Depression

Fig 1 The data indicates that the highest prevalence of depression was observed in certain states, with Punjab, Uttar Pradesh, Madhya Pradesh, and Gujarat having rates of 25.4%, 34.5%, 25.1%, and 26%, respectively. The breakdown by state can provide policymakers with valuable insights into which areas are experiencing a greater burden of depression. This information can be used to identify patterns and develop effective strategies to address the problem.

Table 3 regression analysis was conducted to determine the risk factors associated with depression. Initially, an unadjusted odds ratio was calculated to examine the relationship between the independent variables and the outcome variable. Subsequently a second model was developed to identify the risk factors. Certain variables, such as Age, were grouped into categories (60-70, 71-80, and over 81) to ensure consistency, while Place of Residence was classified as either Rural or Urban, with Urban serving as the reference group. Marital Status was recategorized as Married, Widowed, or Separated, with Married being the reference group. Education was divided into two categories, Illiterate and Literate, with Literate as the reference group. Finally, Wealth Quintile was categorized as Poor, Middle, and Rich, with individuals in the Rich category serving as the reference group.

The table shows that participants living in rural areas had higher odds of developing depression in the unadjusted odds ratio, but it was not statistically significant. However, after adjusting for other variables, the odds of developing depression among rural residents increased to 1.26 (1.15-1.336), which was statistically significant with p<0.05. Additionally, individuals who were widowed or separated had 1.53 (1.40-1.65) times higher odds of developing depression compared to married participants. Low levels of literacy were also significantly associated with the development of depression, with people having higher levels of literacy being less likely to develop depression due to better cognitive abilities and knowledge about health. Furthermore, individuals belonging to the poor wealth quintile had higher odds of developing depression than those in the rich wealth quintile, with the odds decreasing as wealth increased. Consumption of tobacco was a risk factor for depression, with those who consumed tobacco having 1.20 times higher odds of developing depression compared to non-users. Although alcohol consumption was also a risk factor, adjusting for other variables changed the direction of the relationship, with a significant p-value of less than 0.05. Finally, engaging in physical activity was initially a protective factor against depression, but after adjusting for other variables, it became a risk factor with odds of 1.01 (0.94-1.09) and p<0.05. The study discovered that depression was prevalent in rural areas, particularly in states such as Uttar Pradesh, Bihar, Madhya Pradesh, Maharashtra, Himachal Pradesh, Punjab, Uttarakhand, Rajasthan, Arunachal Pradesh, Nagaland, Tripura, Meghalaya, Assam, Jharkhand, Odisha, and Chhattisgarh, where the prevalence was over 75%. In contrast, urban areas in Tamil Nadu, Maharashtra, Madhya Pradesh, Delhi, and Lakshadweep had a depression prevalence of over 75%.

The data provided shows the prevalence of depression across different age groups, literacy statuses, and locations. The prevalence of depression is higher among illiterate individuals compared to literate individuals across all age groups and locations. Depression rates are also generally higher among rural populations compared to urban populations, especially for illiterate individuals.

This information could be useful for healthcare professionals, policymakers, and organizations that aim to address and prevent depression in different populations. For instance, the higher prevalence of depression among illiterate individuals suggests that interventions that focus on improving literacy rates could potentially have a positive impact on reducing depression rates. Additionally, the higher prevalence of depression among rural populations highlights the need for targeted interventions and resources to address mental health in rural areas.

However, it’s important to note that the data only provides information on the prevalence of depression and does not provide any information on the causes or risk factors for depression among these populations. Furthermore, the data only includes a limited set of intersectional variables, and other factors such as socioeconomic status, gender, or race could also be important in understanding depression rates in different populations.

## Discussion

The study participants consisted of 16,737 women aged 60 and above, with participants under the age of 60 excluded from the analysis. The demographic characteristics of the participants were analysed and several important findings were observed. The age group of 60-64 years was the largest with 33.6% of the participants, while the proportion of participants decreased as age increased, with 24.4% of the participants aged over 75 years. This increase in the proportion of older participants is likely due to improved access to healthcare and increased life expectancy.

The majority of the study participants, 63.8%, were from rural areas, and 90.2% were married. A significant proportion, 46.2%, had received basic schooling, while 31.6% had completed at least 10 years of education. The employment status of the participants showed that 60.7% were working, with household income being relatively equal among the different wealth categories. The study also found that a small proportion of the participants, 3.9%, consumed alcohol, while 19% consumed tobacco, and only 12.2% reported regularly exercising. These findings are similar to the results of the ^54^, which reported that a majority of women in India reside in rural areas, have low levels of education and employment, and engage in unhealthy behaviours such as tobacco and alcohol consumption.

It is important to note that these findings provide important insights for policymakers and public health practitioners, highlighting the need for targeted interventions to address the health needs of older women in India, particularly those in rural areas. Further research is needed to understand the impact of demographic and socioeconomic factors on health and well-being in this population, and to develop effective strategies to address the challenges faced by older women in India.

The findings of the study on depression levels and aging are similar to the Global Adult Tobacco Survey (GATS) ^55^, which also found that tobacco use is associated with increased levels of depression. GATS also found that individuals with higher levels of education and those who are employed are less likely to use tobacco and have lower levels of depression. Similarly, the study found that physical activity is associated with lower levels of depression, while GATS found that individuals who are physically active are less likely to use tobacco. However, GATS did not specifically address the relationship between wealth quintiles and depression levels. The study's finding that depression levels were higher among rural participants compared to urban participants is not addressed in GATS. The findings of the study are consistent with other reports regarding depression and risk factors ^56,57^. The study found that depression levels increased with age from 60 to over 75 years, which is similar to other studies that have shown that older adults are at increased risk for depression ^35,58^. Additionally, the study found that depression levels were higher among individuals who consumed alcohol or tobacco, which is also in line with other reports that have linked substance use with increased risk for depression ^59,60^.

However, the study also found some unique findings. For example, the study found that a higher proportion of rural participants reported increased levels of depression compared to urban participants, which is not typically reported in other studies. Additionally, the study found that regularly engaging in physical activity was associated with lower levels of depression, but after adjusting for other variables, physical activity became a risk factor, which is also not a commonly reported finding.

The study's findings on the associations between depression and education, wealth quintile, and marital status are also consistent with other reports. Education, wealth, and marital status have all been linked with mental health, and the study's findings provide further support for these relationships ^27,61^.

Overall, the study adds to the growing body of literature on depression and its risk factors, particularly in older adults. Further research is needed to better the findings of this study that rural areas have a higher prevalence of depression compared to urban areas aligns with previous studies and reports on the topic. This disparity could be due to factors such as lack of access to healthcare, lower socio-economic status, and cultural stigma surrounding mental health in rural areas ^60,62^. However, it's worth noting that while the study found that depression levels were higher in rural areas, there were also some urban areas like Chandigarh, Delhi and Lakshadweep where depression levels were higher. This indicates that the relationship between urbanization and depression levels is complex and not straightforward. Further research is needed to fully understand the factors contributing to the higher levels of depression in both rural and urban areas. understand the complex relationships between depression, demographics, and risk factors. The findings of the study highlight the significance of the place of residence in the development of depression among elderly women, with the rural area being associated with a higher prevalence of depression compared to the urban area. The results show that the prevalence of depression among illiterate women in the rural area was 20.5% for women aged 65-69, and 18.9% for women over 75 years of age, compared to a prevalence of 13% for women over 75 years of age in the urban area. This shows a reduction of 6 points in the prevalence of depression in the urban area compared to the rural area which is in line with the other studies ^23,35^.

Additionally, the study found that the trend of depression prevalence increased with age in the rural area and decreased with age in the urban area. Similarly, the prevalence of depression was found to be higher among literate women over 75 years of age in the rural area (28.6%) compared to urban area (14%) which was just half the prevalence in rural area. The intersectional analysis indicates that the type of place of residence is the major factor for the development of depression, although the underlying cause was not established in the study.

The strength of this study lies in its use of intersectional analysis, which considers the intersection of multiple factors, such as age, literacy, and place of residence, in the development of depression. This provides a more nuanced understanding of the complex relationships between these factors and the development of depression.

However, the weakness of this study is that the underlying cause of the relationship between place of residence and depression was not established. Further research is needed to understand the specific factors that contribute to the higher prevalence of depression in the rural area and the protective effect of urban residency on depression. Additionally, the study is limited by the small sample size and the lack of data on other possible risk factors for depression.

Overall, the study provides important insights into the relationship between place of residence and depression among elderly women and highlights the importance of considering multiple factors in the analysis of mental health outcomes.

Policy implications and recommendations based on this study would include:

1. Addressing the need for mental health services in rural areas: The study highlights that women living in rural areas are more susceptible to depression as compared to their urban counterparts. Thus, it becomes imperative for the government to focus on providing mental health services in rural areas to help reduce the prevalence of depression.
2. Literacy and mental health awareness programs: The study shows that illiterate women are more prone to depression. Hence, promoting literacy and creating mental health awareness programs can help in reducing the incidence of depression.
3. Focus on elderly women: The study highlights that elderly women are more susceptible to depression. The government can focus on creating programs that cater to the needs of elderly women, both in urban and rural areas, to reduce the incidence of depression.
4. Intersectional approach to mental health: The study highlights the importance of an intersectional approach to mental health, which considers multiple factors such as age, literacy, and place of residence. The government can use this approach to develop programs that target specific populations and address the root causes of depression.

Limitations of the study:

1. The sample size: The study may not accurately reflect the prevalence of depression in the entire population due to the limited sample size.
2. Lack of control groups: The study does not have control groups to compare the results, which limits its validity.
3. Cross-sectional design: The study is cross-sectional, which means that it only provides a snapshot of the situation at a particular time. Hence, it cannot be used to establish causal relationships between the variables studied.
4. Uncontrolled variables: The study does not control for other variables that may impact depression such as socioeconomic status, physical health, and access to healthcare services.

## Methodology

The data from the Longitudinal ageing study in India (LASI) Wave 1 (2017–2018) was used to understand the burden of depression among older women above 60 Years in India and to explore the geographic distribution of depression in India. A public domain LASI dataset was obtained from the Gateway to Ageing Portal once the abstract submission was approved. In light of the fact that the data is secondary data, both national and international forums have approved the use of the data. A number of filters were applied to the data to obtain 16,637 samples from women aged 60 and older. Their distribution was calculated using descriptive statistics by gender, age, place of residence, and education status. The data from the Longitudinal ageing study in India (LASI) Wave 1 (2017–2018) was used to understand the burden of depression among the older women above 60 Y in India and to explore the geographic distribution of the depression in India. LASI is the first longitudinal dataset in India to provide a reliable basis for designing policies and programmes for the older population's social, health, and economic wellbeing. LASI uses Computer-Assisted Personal Interview (CAPI) technology, internationally Harmonized/ Gold Standard Survey Protocol, Comprehensive Range of Biomarkers. Multistage stratified area probability cluster sampling design is used for selecting the representative sample in each stage. The eligibility criteria was older adults aged 45 Years and above (including spouses irrespective of age).The eventual unit of observation of LASI was LASI-eligible household (LEH) with at least one-member age 45 and above. LASI adopted a multistage stratified area probability cluster sampling design to arrive at the eventual units of observation. All the 30 Indian States and six Union Territories were selected for the survey. The states were further divided in to Districts, Sub districts, Talukas, Tehsils and Blocks. The samples were selected in four stages, where in the first state was for selection of Primary Sampling Unit (PSU) and second and third stage was for selection of Secondary Sampling Unit (SSU), and fourth stage was for selection of households. For assessing depression, the tools used are Centre for Epidemiologic Studies Depression (CES-D) to find the symptoms of depression and Composite International Diagnostic Interview-Short Form (CIDI-SF) scale to diagnose major depression. For more details please refer to LASI India Report 2020 at ^29^.

**Figure 4:**
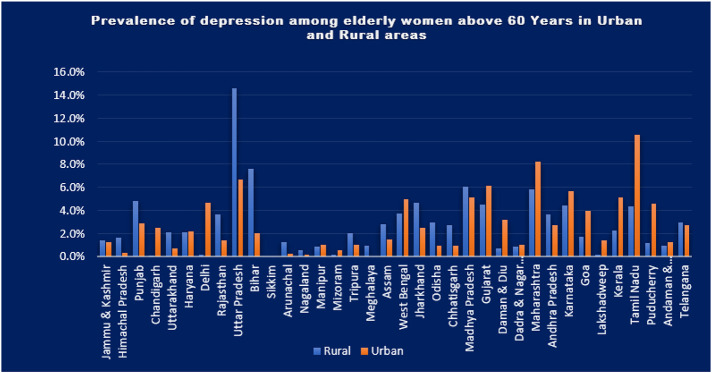
Bar graph showing the prevalence of depression among the women aged 60 and above in Urban and Rural Area Variation

It is a cross sectional study aimed to explore the factors responsible to determine the prevalence among elderly women of 60 and above in India using the intersectional analysis. We were able to explore the intersecting patterns between different variables and how people are positioned simultaneously and position themselves in different multiple categories based on the type of place of residence, age and level of education. Through the study we further aim to determine the prevalence of depression among elderly female in the age group of 60 in different states using the Chloropleth map. Binary Logistic Regression was used to estimate the prime factors associated with depression since depression was a binary yes or no variable. Odds Ratio was calculated using clinical and demographic variables. Unadjusted odd ratio was obtained through the first regression model to control other variables. Our second regression model takes into account other variables depicted in Fig:4 to determine the risk factors for depression with a confidence interval of .001. Using intersectional analysis, we explored how people are positioned simultaneously according to their place of residence, their age, and their level of education, as well as how they position themselves in different multiple categories and if conditional probability of depression exists in all categories. Our next step was to compute the prevalence of depression at the state level. To understand the distribution of depression prevalence among Indian states and Union Territories, we have plotted the Choropleth map using the GeoDa software.

## Conclusion

In conclusion, the study analysed the prevalence of depression among elderly women aged 65 to 69 and more than 75 in rural and urban areas. The results showed that the prevalence of depression among rural elderly women was higher compared to urban elderly women. However a disparity among states was found. The trend of prevalence increased with age among rural women and decreased with age among urban women. Additionally, the intersectional analysis showed that the type of place of residence was the major factor for the development of depression.

The study highlights the need for mental health policies and interventions to address the higher prevalence of depression among elderly rural women. This may involve providing access to mental health services, creating community support systems, and raising awareness about mental health in rural areas. Furthermore, there is a need for further research to understand the underlying causes of depression among rural elderly women.

While the intersectional analysis provides a useful insight into the complex relationship between demographic factors and mental health, it is important to acknowledge its limitations. The study did not establish the cause of depression, and it was beyond the scope of this analysis. Moreover, the study relied on self-reported data, which may be subject to bias.

In summary, the study provides valuable information on the prevalence of depression among elderly women in rural and urban areas and the importance of considering the intersection of demographic factors in understanding mental health. The results of this study can inform policy and practice to improve mental health outcomes among elderly women, especially those living in rural areas.

## Figures and Tables

**Figure 2:**
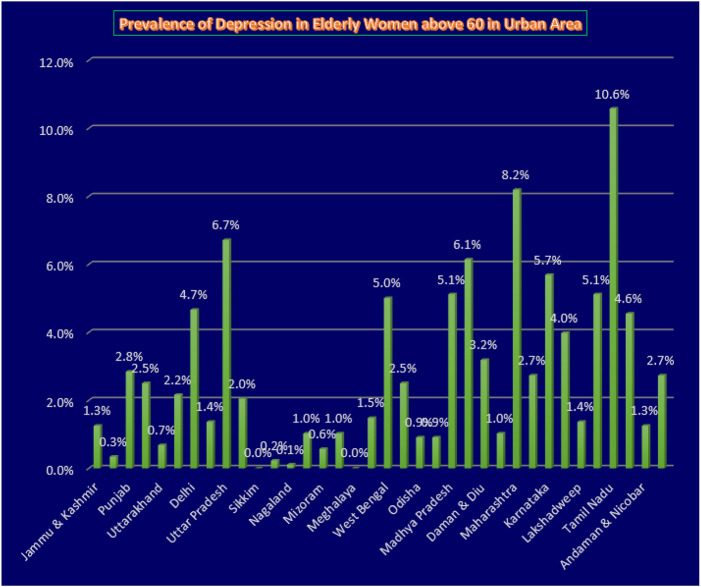
Bar graph showing the prevalence of depression among the women aged 60 and above in Urban Area

**Figure 3:**
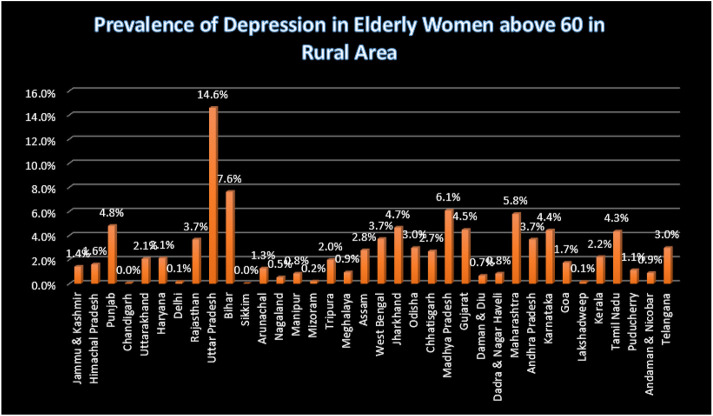
Bar graph showing the prevalence of depression among the women aged 60 and above in Rural Area

**Figure 5:**
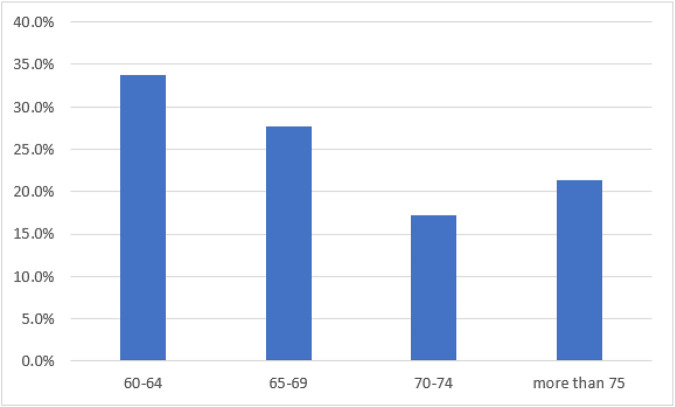
Bar graph showing the prevalence of depression among the women aged 60 and above

**Table 1:** Demographic characteristics of the study participants.

		N	Percentage
Age	60-64	4183	25.3%
	65-69	5142	31.1%
	70-74	3240	19.6%
	more than 75	3985	24.1%
Place of residence	Rural	10763	65.1%
	Urban	5770	34.9%
Current marital Status of the person	Currently Married	6325	43.5
	Widowed	7853	54%
	Divorced/separated/Deserted	372	2.5%
Education	less than 5 years	3231	22.2%
	5 to 9 years	6724	46.2%
	more than 10 years	4599	31.6%
Currently working N= 7380	Yes	2427	32.9
	No	4953	67.1
Household income	Poorest	3521	22.8%
	Poorer	3700	22.6%
	Middle	3225	19.7%
	Richer	3012	18.4%
	Richest	2521	15.4%
Alcohol	Yes	621	4.3%
	No	13932	96.1%
Tobacco	Yes	3364	23.1%
	No	11189	76.9%
physical activity	Yes	1294	8.9%
	No	13259	91.1%

**Table 3-:** Multivariate logistic regression of sociodemographic and associated risk factors The model was obtained as below Y= β_0_+ β_1_X_1_+ β_2_X_2_+Epsion.

	Un-Adjusted Odds Ratio						
Adjusted Odds Ratio							
Name of the Variable	Categories of the variable	Odds Ratio	Confidence Interval	P value	Odds Ratio	Confidence Interval	P value
**Age**	60-70	1					
	71-80	0.97	0.89-1.05	0.62	0.82	0.74-0.88	0.001
	More than 81	0.83	0.76-0.90	0.001	0.57	0.51-0.62	0.001
**Place of Residence**	Rural	1.32	1.23-1.44	0.71	1.26	1.15-1.336	0.001
	Urban	1		1			
**Marrital Status**	Married	1		1			
	Widowed/Seperated	1.70	1.56-1.85	0.001	1.53	1.40-1.65	0.001
**Education**	Illiterate	1.23	1.10-1.37	0.002	1.03	0.93-1.15	0.06
	Literate	1		1			
**Currently working status**	Yes	1		1			
	No	1.20	1.12-1.30	0.001	1	.91-1.07	0.048
**Wealth Quintile**	Poor	1.16	1.03-1.30	0.000	1.20	1.09-1.36	0.001
	Middle	1.09	0.97-1.21	0.010	1.06	.95-1.19	0.000
	Rich	1		1			
**Tobacco consumption**	Yes	1.20	1.10-1.30	0.001	1.20	1.10-1.30	0.001
	No	1					
**Alcohol consumption**	Yes	1.16	1.04-1.29	0.000	1.10	.98-1.22	0.47
	No	1					
**Physical Activities**	Ys	0.94	0.83-0.98	0.010	1.01	0.94-1.09	0.000
	No	1		1			

**Table 4:** The occurrence or frequency of depression in relation to rural and urban areas.

Place of residence				
State	Rural		Urban	
	Count	Column N %	Count	Column N %
1.Jammu & Kashmir	30	1.4%	11	1.3%
2.Himachal Pradesh	34	1.6%	3	0.3%
3.Punjab	102	4.8%	25	2.8%
4.Chandigarh	1	0.0%	22	2.5%
5.Uttarakhand	44	2.1%	6	0.7%
6.Haryana	45	2.1%	19	2.2%
7.Delhi	3	0.1%	41	4.7%
8.Rajasthan	78	3.7%	12	1.4%
9.Uttar Pradesh	310	14.6%	59	6.7%
10.Bihar	162	7.6%	18	2.0%
11.Sikkim	0	0.0%	0	0.0%
12.Arunachal	27	1.3%	2	0.2%
13.Nagaland	11	0.5%	1	0.1%
14.Manipur	18	0.8%	9	1.0%
15.Mizoram	4	0.2%	5	0.6%
16.Tripura	42	2.0%	9	1.0%
17.Meghalaya	20	0.9%	0	0.0%
18.Assam	59	2.8%	13	1.5%
19.West Bengal	79	3.7%	44	5.0%
20.Jharkhand	99	4.7%	22	2.5%
21.Odisha	63	3.0%	8	0.9%
22.Chhatisgarh	57	2.7%	8	0.9%
23.Madhya Pradesh	129	6.1%	45	5.1%
24.Gujarat	95	4.5%	54	6.1%
25.Daman & Diu	14	0.7%	28	3.2%
26.Dadra & Nagar Haveli	18	0.8%	9	1.0%
27.Maharashtra	123	5.8%	72	8.2%
28.Andhra Pradesh	78	3.7%	24	2.7%
29.Karnataka	94	4.4%	50	5.7%
30.Goa	37	1.7%	35	4.0%
31.Lakshadweep	3	0.1%	12	1.4%
32.Kerala	47	2.2%	45	5.1%
33.Tamil Nadu	92	4.3%	93	10.6%
34.Puducherry	24	1.1%	40	4.6%
35.Andaman & Nicobar	19	0.9%	11	1.3%
36.Telangana	63	3.0%	24	2.7%

**Table 5:** Intersectional- analysis based on the level of education, place of residence, and age.

Variable of Intersectional analysis	Prevalence of Depression
Uneducated, Rural, age 60-64	17%
Uneducated, Urban, age 60-64	16%
Uneducated, Rural, age 65-69	21%
Uneducated, Urban, age 65-69	14%
Uneducated, Rural, age 70-74	20%
Uneducated, Urban, age 70-74	18%
Uneducated, Rural, age more than 75	19%
Uneducated, Urban, age more than 75	13%
Educated, Rural, age 60-64	13%
Educated, Urban, age 60-64	11%
Educated, Rural, age 65-69	15%
Educated, Urban, age 60-64	11%
Educated, Rural, age 70-74	10%
Educated, Urban, age 70-74	10%
Educated, Rural age more than 75	29%
Educated, Urban, age more than 75	13%

## Data Availability

The data that support the findings of this study are available in Gateway to Ageing at https://g2aging.org/downloads, reference number “R01 AG030153”. These data were derived from the following resources available in the public domain: https://g2aging.org/login&r=%5Eq%5E https://g2aging.org/?section=login&r=^q^section=downloads https://www.iipsindia.ac.in/content/lasi-wave-i
